# Reduced Renal CSE/CBS/H2S Contributes to the Progress of Lupus Nephritis

**DOI:** 10.3390/biology12020318

**Published:** 2023-02-16

**Authors:** Xuan Wang, Tao Lin, Yifei He, Yueyuan Zhou, Yi Peng, Weiru Zhang, Xin Ni

**Affiliations:** 1 Department of General Medicine, Xiangya Hospital, Central South University, Changsha 410008, China; 2 International Collaborative Research Center for Medical Metabolomics, Xiangya Hospital, Central South University, Changsha 410008, China; 3 National Clinical Research Center for Geriatric Disorders, Xiangya Hospital, Central South University, Changsha 410008, China; 4 Department of Rheumatology and Immunology, Xiangya Hospital, Central South University, Changsha 410008, China

**Keywords:** H2S, CSE, CBS, lupus nephritis, STAT1, RELA, T cells

## Abstract

**Simple Summary:**

Lupus nephritis is a severe and fatal immune-associated nephritis. The molecular mechanisms underlying lupus nephritis development remain largely unknown. Renal hydrogen sulfide can be produced mainly via two synthetases and is involved in many pathological and physiological processes. Herein, we sought to investigate the roles of renal hydrogen sulfide and its synthetase in lupus nephritis pathogenesis. We found that the expression of two hydrogen sulfide synthetases was downregulated in renal tissues of lupus nephritis patients and lupus mice, which were associated with poor renal outcomes. Moreover, exogenous hydrogen sulfide attenuated renal damage in two mouse models with lupus nephritis. In addition, we further confirmed the increase of renal key transcription factors and T-lymphocyte infiltration in lupus nephritis, which were mitigated after treatment with hydrogen sulfide donors. Our composite data indicated that reduced renal two hydrogen sulfide synthetases contribute to the progress of lupus nephritis, and exogenous hydrogen-sulfide-attenuated renal damage of lupus nephritis may partly be through inhibiting the expression of T-lymphocyte-associated transcription factors.

**Abstract:**

The molecular mechanisms underlying lupus nephritis (LN) pathogenesis are not fully understood. Hydrogen sulfide (H2S) is involved in many pathological and physiological processes. We sought to investigate the roles of H2S in LN pathogenesis. H2S synthase cystathionine–lyase (CSE) and cystathionine–synthetase (CBS) expression was downregulated in renal tissues of patients with LN and their levels were associated with LN’s prognosis using the Nephroseq database. Reduced CSE and CBS protein expression in kidney tissues of LN patients and MRL/lpr mice were confirmed by immunohistochemistry. CSE and CBS mRNA levels were reduced in MRL/lpr and pristine- and R848-induced lupus mice. Given that H2S exerts an anti-inflammatory role partly via regulating inflammatory transcription factors (TFs), we analyzed hub TFs by using a bioinformatics approach. It showed that STAT1, RELA, and T-cell-related signaling pathways were enriched in LN. Increased STAT1 and RELA expression were confirmed in renal tissues of LN patients. Treatment of MRL/lpr and pristine mice with H2S donors alleviated systemic lupus erythematosus (SLE) phenotypes and renal injury. H2S donors inhibited RELA level and T-cell infiltration in the kidneys of MRL/lpr and pristine mice. Our data indicated that CSE/CBS/H2S contributes to LN pathogenesis. Supplementation of H2S would be a potential therapeutic strategy for LN.

## 1. Introduction

Systemic lupus erythematosus (SLE) is a complex autoimmune disease with a wide range of clinical manifestations. It is characterized by positive tests for auto-antibodies, complement activation, and immune-complex deposition, thereby resulting in the inflammation of multiple organs. SLE can affect many organs, of which one of the most important is the kidney [1,2]. It has been reported that lupus nephritis (LN) develops in 30–60% patients with SLE. LN is a major risk factor for overall morbidity and mortality in SLE, and 10% of patients with LN will develop end-stage renal disease (ESRD) [3]. Management of SLE has greatly been improved in the past decades. The current treatment strategy for LN includes potent anti-inflammatory and immunosuppressive therapies for LN of class II–IV [3,4]. However, many patients still develop chronic kidney disease (CKD) and ESRD after treatment, which is, at least in part, attributed to the fact that the molecular mechanisms underlying LN pathogenesis are not fully understood [5].

The pathogenesis of LN involves the extrarenal etiology of systemic lupus and the intrarenal etiology of LN. The extrarenal pathogenesis is associated with a loss of tolerance and the presence of antinuclear antibodies, as well as activated innate and adaptive immunity caused by nucleic acids released from apoptotic immune cells [6]. The intrarenal etiology involves antibody binding to multiple intrarenal autoantigens, immune complexes depositing in the mesangium or the subendothelial and subepithelial spaces or in peritubular capillaries, intrarenal activation of toll-like receptor (TLR) signaling, and recruitment of different leukocyte subsets [1,6]. Nevertheless, the major pathological manifestations of LN are inflammation and subsequently maladaptive tissue repair in the kidney [6,7]. Although there are several hypotheses regarding the development and progress of local inflammation in the kidney of SLE, the key links or molecules that promote the local inflammatory response of the kidney in LN are still unclear.

Hydrogen sulfide (H2S) is the third most important gas signal molecule after nitric oxide and carbon monoxide [8,9]. H2S is synthesized from L-cysteine mainly by the enzymes cystathionine-γ-lyase (CSE) and cystathionine-β-synthetase (CBS) via the transsulfuration pathway. In mammalian organisms, CSE and CBS are biologically essential and distributed in various tissues. H2S is implicated in many physiologic and pathologic processes including vasodilation, angiogenesis, and inflammation [10]. Of note, H2S is involved in many processes of inflammatory responses in various tissues, such as the modulation of the functions of immune cells, regulation of chemokine and cytokine production in the tissues, and modulation of some signaling pathways linked to inflammation, such as MAPK and NF-κB [11,12]. H2S can exert either anti-inflammatory or pro-inflammatory effects depending on its concentration, tissue specificity, and physiological and pathophysiological condition [9]. Both CSE and CBS are expressed in the kidney including tubules and glomeruli. Some studies have demonstrated that the CSE/CBS/H2S system is involved in the pathological processes of acute kidney diseases (AKD) including ischemia/reperfusion renal injury and nephrotoxic damage [13,14,15,16], as well as CKD, such as renal fibrosis and diabetic nephropathy [17,18]. To our knowledge, whether the CSE/CBS/H2S system is involved in the progress of LN has not been investigated, although Han et al. reported that H2S supplementation inhibits the proliferation of circulatory lymphocytes in SLE patients.

Recent studies highlighted the critical role of transcription factors (TFs) in modulating the immune response and inflammation in kidney diseases, including LN [19,20,21]. The role of TFs might be context-dependent and vary among different renal injuries. For example, acute kidney injury is associated with transcription factors such as NRF2 that regulate energy metabolism in renal tubular epithelial cells [22], while the expression and activation of Hif-1α are associated with hypertensive nephropathy [23]. In addition, we have previously found that H2S regulates the expression of transcription factor STAT6 in renal injury and fibrosis [24]. Thus, it is of great interest to understand the association of the CSE/CBS/H2S system with the key TFs in LN pathogenesis.

The aims of the present study were to investigate whether the CSE/CBS/H2S system contributes to the development and progress of LN, and further analyze the association of CSE/CBS/H2S with TFs. At first, we analyzed the expression levels of CSE and CBS in the renal tissues of SLE patients with LN and the association of CSE and CBS levels with the prognosis of LN patients using the Nephroseq database. Then, we examined the CSE and CBS expression levels in the kidney tissues of LN patients and renal tissues of several animal models of SLE. Further, we analyzed the association of CSE and CBS expression levels with TFs linked to LN in renal tissues using a bio-informatics approach. Finally, we investigated the effects of supplementation of H2S donors on overall phenotypes of SLE and LN, CSE and CBS levels, and expression of hub genes in two SLE mouse models. We revealed that CSE and CBS were downregulated in renal tissues in LN patients and SLE mouse models. Exogenous H2S could alleviate kidney injury, inhibit the expression of NF-κB (p65), and reduce T-cell infiltration in kidney. Our data provide an update on the pathogenic mechanisms that lead to LN and immediately highlight the rationale for the latest and novel treatment strategies.

## 2. Materials and Methods

### 2.1. Human Tissues

CSE and CBS expression levels were measured in collected renal biopsies from patients hospitalized at Xiangya Hospital of Central South University, Changsha, Hunan, China. All patients gave informed consent for this study and the publication of relevant images of kidney tissue. The ethics committee of the Xiangya Hospital Central South University approved all protocols. Overall, 6 LN patients who completed renal needle biopsy were enrolled, and five kidneys underwent nephrectomy, which renal pathology indicated to be normal or exhibiting minimal change, and were used as controls.

### 2.2. Pristane-Induced Lupus Mice

Female Balb/c mice at 8 weeks old were purchased from SLAC laboratories (Changsha, China). During the study period, the mice were placed in the Animal Breeding Center of Central South Xiangya Medical College and were free to obtain food and water. All animal protocols are reviewed and approved by the Animal Welfare Committee of Central South University. The mice were divided into three groups: control group, pristane group, and pristane with NaHS treatment group. Pristine was purchase from Sigma-Aldrich (St. Louis, MO, USA). Each mouse in pristane group received 0.5 mL pristane (i.p) once at 8 weeks old. The mice in pristane with NaHS treatment group received 0.5 mL pristane (i.p) once at 8 weeks old, and then received NaHS (i.p) at 25 μg/kg at 20 weeks old every day until 24 weeks old. The control mice were treated with saline in same way. The mice were sacrificed, and the sample was collected at the age of 24 weeks.

### 2.3. MRL/lpr Mice

Female MRL/lpr lupus mice and MRL/mpj control mice were purchased from SLAC laboratory (Shanghai, China). At the age of 14 weeks, MRL/lpr mice were injected by i.p. with saline, and GYY4137 (10 mg/kg/day), respectively. The GYY4137 treated mice were sacrificed at the age of 16 weeks, and blood and tissue samples of mice were collected. Furthermore, another three groups of MRL/lpr mice have been divided: injected with saline, NaHS (30 mg/kg/day), and NaHS (60 mg/kg/day) at the age of 12 weeks, respectively. The NaHS-treated mice were sacrificed at the age of 20 weeks. Urine protein, urine creatinine, serum ALB, and serum kidney index of lupus mice were detected by kit from Nanjing Jiancheng Biological Company following the instructions.

### 2.4. R848-Induced Lupus Mice

Female Balb/c mice at 8 weeks old were purchased from SLAC laboratories (Changsha, China). The mice were divided into control group and R848 group. The topical TLR-7 agonist resiquimod R848 (Selleck) was dissolved in DMSO, and 100 μg of R848 in 100 μL of DMSO was administered to the ear 3 times weekly [25]. The control mice received DMSO. The mice were sacrificed at the age of 16 weeks. 

### 2.5. HE Staining, Immunohistochemical (IHC) Staining, and Immunofluorescence (IF) Staining

Mice kidneys and joints were fixed with 10% buffered formalin, embedded in paraffin, and then sectioned for hematoxylin and eosin staining. Mouse joints were decalcified before sectioning. Human and mouse renal samples in paraffin or embedding were sliced into 4 μm sections at Xiangya Hospital Central South University. For IHC staining, dewaxing and antigen retrieval were performed by microwaving P100 in EDTA for 20 min. The slides were treated with hydrogen peroxide at room temperature for 20 min, and blocked with goat serum for 1 h. The paraffin slides were incubated with anti-CSE antibody (Abcam, Cambridge, MA, USA, ab6604), anti-CBS antibody (Santa Cruz Biotechnology, Santa Cruz, CA, USA, sc-133154), anti-STAT1 antibody (Servicebio, GB111363), anti-p-STAT1 (Abcam, Cambridge, MA, USA, ab109461) antibody, anti-p65 (Cell Signaling Technology, Danvers, MA, USA, 8242T) antibody, anti-p-p65 (Cell Signaling Technology, Danvers, MA, USA, 3033T) antibody, anti-CD45 (Cell Signaling Technology, Danvers, MA, USA, 70257S) antibody, and anti-CD3 (Proteintech, Wuhan, Hubei, PRC, 17617-1-AP) antibody in a humidified chamber at 4 ℃ overnight. After washing in PBS, slides were incubated with secondary antibody for 30 min at 37 ℃. DAB and hematoxylin were used as chromogens.

For IF staining, the frozen slides returned to room temperature and were then fixed with 4% paraformaldehyde for 30 min. After three washes with PBS, the slides were as blocked and incubated as IHC. Mouse anti-α-SMA antibody (Santa Cruz Biotechnology, Santa Cruz, CA, USA, sc-53142), rabbit anti-CSE (Abcam, Cambridge, MA, USA, ab136604) antibody, and rabbit anti-CBS (Proteintech, Wuhan, Hubei, PRC, 14787-1-AP) antibody were used for the first antibody in IF staining. Then, slides were incubated with conjugated fluorescent anti-mouse or anti-rabbit secondary antibody for 30 min and nuclear staining reagent DAPI for 10 min. Other reagents of staining were purchased from ZSGB-BIO. The method and procedure of HE and immunostaining were performed as previously described [26].

### 2.6. Microarray Data and Identification of Differentially Expressed Genes (DEGs)

Microarrays in the Nephroseq database (www.nephroseq.org, accessed on 6 September 2022), namely, Berthier lupus Glom and Berthier lupus TubInt which combined both glomerular and tubulointerstitial samples [27], have been used to analyze the transcriptome of CSE and CBS in microdissected renal biopsy samples. In addition, the correlation between the expression the H2S and the index of renal damage has been analyzed. The renal tissue gene expression profiles of GSE32591 and GSE113342 from LN patients and normal controls were downloaded from the GEO database. The GSE32591 dataset contained 93 samples, including 64 samples with LN (32 tubulointerstitium and 32 glomeruli) and 29 living donors (LD, 15 tubulointerstitium and 14 glomeruli) [27]. The GSE113342 dataset contained 72 samples, including 56 samples with LN (28 tubulointerstitium and 28 glomeruli) and 16 normal controls (NC, 10 tubulointerstitium and 6 glomeruli) [28]. Then, the annotation document of corresponding platforms was used to annotate the gene expression profile in each dataset. Finally, the matrix with row names as sample names and column names as gene symbols was obtained for subsequent analysis. DEGs between LN and LD/NC biopsy samples were defined by adjust *p* < 0.05 and |log fold change (FC)| >1.0 using the “limma” package in RStudio (version 2022.07.0 + 548). The DEGs common to the two GEO databases were analyzed by Jvenn, an interactive Venn diagram viewer [18]. Human TF genes and TF cofactor genes (downloaded from Human TFDB, http://bioinfo.life.hust.edu.cn/HumanTFDB#!/, accessed on 20 september 2022, HUST, Wuhan, Hubei, PRC), common to DEGs, namely, DETFGs and DEG of TF cofactors, have also been collected.

### 2.7. Functional Enrichment and Protein–Protein Interaction (PPI) Analysis

Based on DETFGs in the tubulointerstitium and glomeruli, the Kyoto Encyclopedia of Genes and Genomes (KEGG) database has been used to understand high-level functions and biological systems in differential TFs of LN. RStudio was used for pathway annotations. Statistical significance was set at *p* < 0.05. A PPI network in DETFGs of tubulointerstitium and glomeruli was established using the STRING (version 10.0) online search tool. The PPI network was visualized using Cytoscape (version 3.7.2), and one type of assignment module, cytohHubba, was used for sorting top genes by degree.

### 2.8. ROC Analysis and Profile Infiltrating Immune Cells with ImmucellAI in the Kidney of LN

ROC analysis was used to test the diagnostic value of the differential transcription factors in RStudio. The area under the curve (AUC) was presented with 95% confidence intervals (CI) of DETFGs in tubulointerstitium and glomeruli, respectively. The GSE112943 dataset has been used as a validation dataset to verify the expression of key differential transcription factor genes. To assess the expression changes in immune cells and obtain the proportion of various types of immune cells from tubulointerstitium and glomeruli, we employed the online ImmuCellAI (http://bioinfo.life.hust.edu.cn/ImmuCellAI#!/, accessed on 20 september 2022, HUST, Wuhan, Hubei, PRC) [29]. The more enormous amount of data in the database, GSE32591 series matrix txt format files, were downloaded from the NCBI GEO website, and the expression data were different in 24 immune cells in ImmuCellAI (18 T cells and 6 other immune cell types including B cells, NK cells, monocytes, macrophages, neutrophils, and dendritic cells). The DETFGs and differential immune cell types in the kidney in GSE32591 were used for this correlation analysis. We used RStudio for correlation analysis and statistical significance was set at *p* < 0.05.

### 2.9. Real-Time qPCR Analysis

The mice’s kidney was frozen immediately in liquid nitrogen after separation. Total RNA was isolated using Trizol (Life Technologies, Gaithersburg, MD, USA), and equal amounts (500–1000 ng) were reverse transcribed using a cDNA synthesis kit (GeneCopoeia, Rockville, MD, USA). Quantitative PCR on a 1:4 dilution of the cDNA was then performed using the QuantStudio Real-Time PCR detection biosystem. Gene expression was normalized to GAPDH or β-actin. The protocol for the qPCR array assay was similar to common practice. As previously described, the mRNA expression of CSE, CBS, and β-actin was detected by qPCR [26]. The primers were as follows:

Forward of mouse β-actin primer: CACTGTCGAGTCGCGTCC.

Reverse of mouse β-actin primer: TCATCCATGGCGAACTGGTG.

Forward of mouse CSE primer: AGATGCCACCCTCCTGAAGTACC.

Reverse of mouse CSE primer: TTGCTGCCACCATTACGATTACCC.

Forward of mouse CBS primer: TGTGAAGATGGCTCTGCTGG.

Reverse of mouse CBS primer: CCAGGTACATCTGCTTGGGG.

### 2.10. Statistical Analysis

The data are expressed as the means ± SEM. GraphPad Prism (version 8.3.0) software was used for statistical analysis. Two-group Student t tests (paired or unpaired as appropriate) were applied. Differences between the means of multiple groups were compared by the one-way analysis of variance (ANOVA), followed by a Tukey multiple comparison test. A value of *p* < 0.05 was considered statistically significant.

## 3. Results

### 3.1. CSE and CBS Are Downregulated in Renal Tissues of Patients with LN and Are Associated with LN’s Prognosis

**Figure 1:**
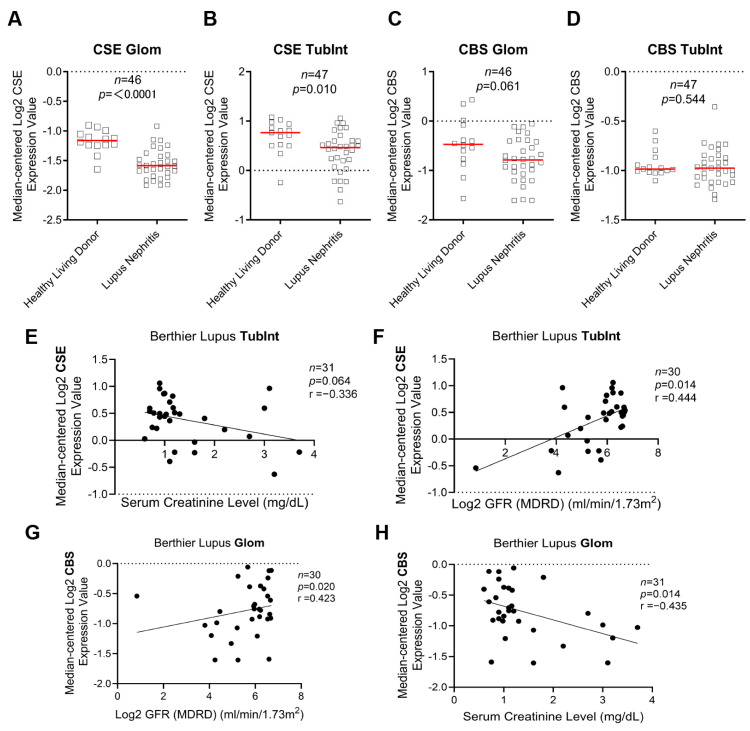
Downregulation of hydrogen sulfide synthetase in the kidneys of lupus nephritis patients was associated with poor renal prognosis. (**A**,**B**) Gene expression of renal CSE in Glom and TubInt of healthy living donors and LN patients (Nephroseq database). (**C**,**D**) Gene expression of renal CBS in Glom and TubInt of healthy living donors and LN patients. (**E**,**F**) Correlation of renal TubInt CSE gene expression with serum creatinine and GFR in patients with LN in Nephroseq database. (**G**,**H**) Correlation of renal Glom CBS gene expression with serum creatinine and GFR in patients with LN in Nephroseq database.

Firstly, through the Nephroseq database (Berthier lupus seq database), we found the mRNA expression of H2S synthetase CSE (also known as CTH in the Nephroseq database) was downregulated in the glomeruli (Glom) and tubulointerstitium (TubInt) of LN, and CBS was downregulated in Glom (Figure 1A–D). The gene expression of TubInt CSE is negative with the serum creatinine and positive with the log2 glomerular filtration rate (GFR) value in patients with LN (Figure 1E–F). The correlation between renal function index and expression of Glom CBS showed the same trend (Figure 1G–H). However, Glom CSE and TubInt CSE showed no statistical difference with the renal function index (data not shown).

### 3.2. CSE and CBS Levels Are Reduced in the Renal Tissue of LN Patients and Lupus Mice

**Figure 2:**
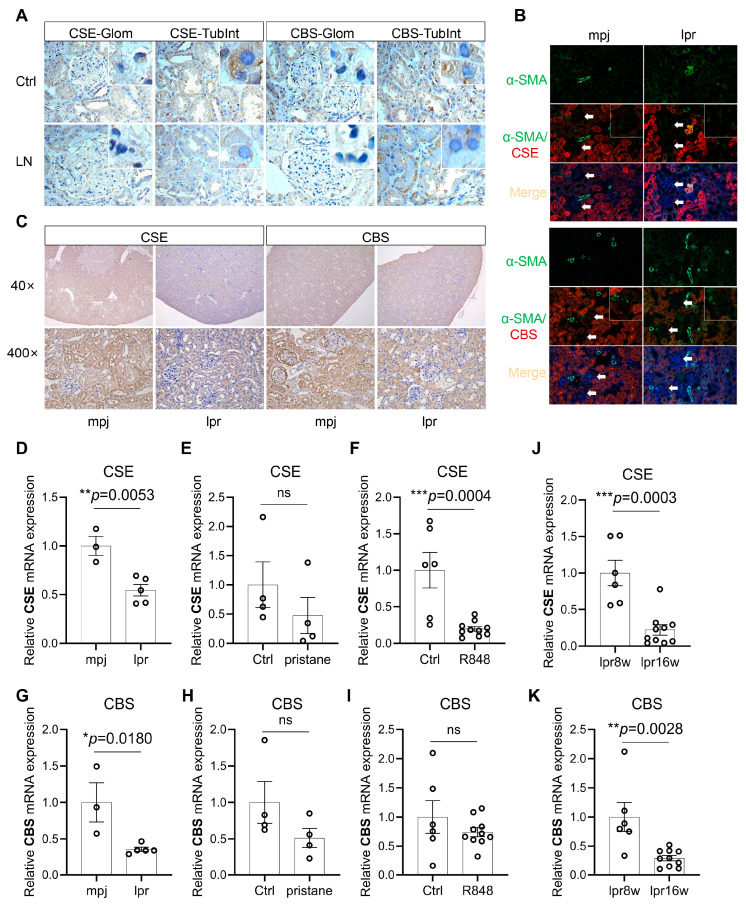
CSE and CBS were decreased in the kidney tissue of lupus mice. (**A**) Representative immunohistochemical staining images of CSE and CBS from human renal-puncture tissue (400×). Glom: glomeruli; TubInt: tubulointerstitium; *n*: total number of data; Ctrl: normal or minimal change kidney of the donor. (**B**) Representative immunofluorescence staining images of CSE, CBS, DAPI, and αSMA (a marker of glomerular mesangial cells and renal interstitial vascular smooth muscle cells) from the kidney of MRL/mpj and MRL/lpr mice (200×); the white arrows and enlarge box in the upper right corner of pictures refer to the glomerulus. (**C**) Representative immunohistochemical staining images of CSE and CBS from the kidney of MRL/mpj and MRL/lpr mice (40× and 400×). (**D**) mRNA expression of CSE in 16-week-old MRL/mpj and MRL/lpr mice. (**E**) mRNA expression of CSE in control Balb/c and pristane-induced Balb/c mice. (**F**) mRNA expression of CSE in control Balb/c and R848-induced Balb/c mice. (**G**) mRNA expression of CBS in 16-week-old MRL/mpj and MRL/lpr mice. (**H**) mRNA expression of CBS in control Balb/c and pristane-induced Balb/c mice. (**I**) mRNA expression of CBS in control Balb/c and R848-induced Balb/c mice. (**J**,**K**) mRNA expression of CSE and CBS in 8-week-old (pre-diseased) and 16-week-old (severely diseased) MRL/lpr mice. *, *p* < 0.05; **, *p* < 0.01; ***, *p* < 0.001.

Next, we validated the expression levels of CBS and CSE in kidney tissues of SLE patients with LN. As shown in Figure 2A, CSE and CBS were highly expressed in the TubInt in control patients. They were robustly reduced in the TubInt of LN patients. We also observed the CSE and CBS expression in the renal tissues of MRL/lpr mice, with a-SMA as a marker of mesangial cells in Glom and vascular smooth muscle in TubInt. IHC and immunofluorescence staining showed that CSE and CBS were highly expressed in TubInt; the positive staining of CSE and CBS was identified in the glomeruli of MRL/mpj mice. MRL/lpr mice showed a reduced CSE and CBS expression in the glomeruli and TubInt compared with MRL/mpj mice (Figure 2B,C). We further investigated the CSE and CBS expression in three SLE mouse models. As shown in Figure 2D–I, CSE and CBS mRNA levels were significantly reduced in the kidney of 16-week-old MRL/lpr mice, pristane-induced lupus mice, and R848-induced lupus mice, even though the expression in pristane-induced mice showed no significance. In MRL/lpr mice, CSE and CBS mRNA levels were decreased with the lupus progress as evidenced by the fact that they were further reduced at 16 weeks old compared with 8 weeks old (Figure 2J–K).

### 3.3. RELA (p65) and STAT1 Are Hub TFs in the Kidney of LN Patients and CSE and CBS Levels Are Correlated with STAT1 and RELA Levels

**Figure 3:**
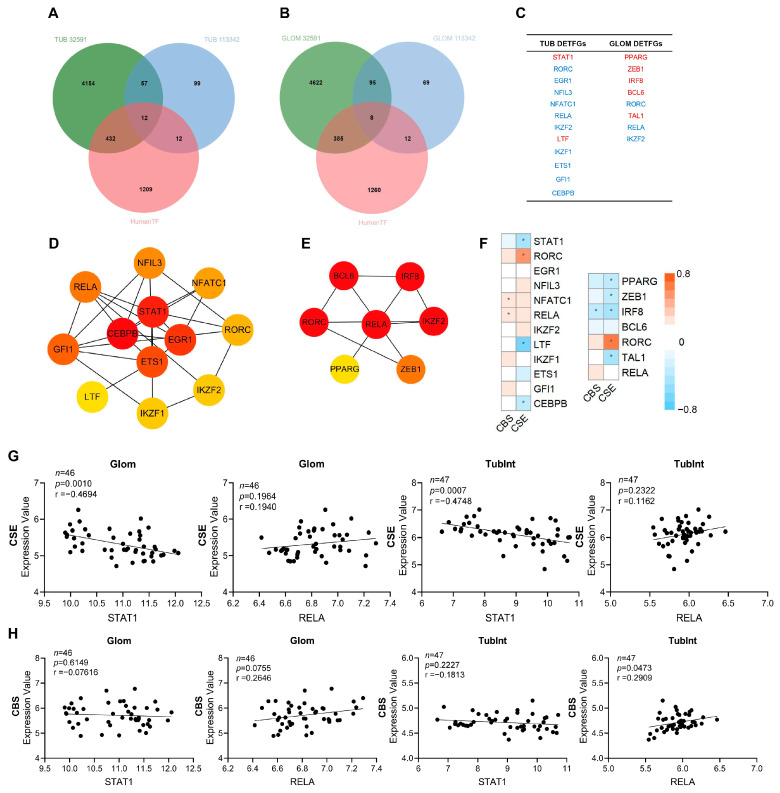
Screen of key TFs in renal tissue of patients with LN and its correlation with CBS and CSE. The Venn diagram of the differential TFs in the tubulointerstitium (**A**) and glomeruli (**B**), based on GSE32591, GSE113342, and human TFDB. (**C**) Red represents upregulation and blue represents downregulation for the differentially expressed TF genes (DETFGs) in the tubulointerstitium and glomeruli. (**D**) The interaction of hub transcription factors in the tubulointerstitium: the redder the color, the higher the degree through modules cytohHubba for sorting top hub genes. (**E**) The interaction of hub transcription factors in the glomeruli. (**F**–**H**) Correlation analysis between gene expression of CSE, CBS, and DETFGs. TUB/TubInt, tubulointerstitium; GLOM, glomeruli; *, *p* < 0.05.

We used “limma” to analyze the differential genes in the renal tissues of patients with LN and normal controls. From the GSE32591 and GSE113342 datasets, 69 DEGs in the tubulointerstitium (Figure 3A) and 103 DEGs in the glomeruli (Figure 3B) were identified. Further co-analysis with the human TF database (Table S1) indicates that 12 TFs in the tubulointerstitium and eight TFs in the glomeruli were significantly altered in LN. Two upregulated TFs (STAT1 and LTF) and 10 downregulated TFs (RORC, EGR1, NFIL3, NFATC1, RELA (p65), IKZF1, IKZF2, ETS1, GFI1, and CEBPB) were identified in the tubulointerstitium of LN (Figure 3C). While five TFs including PPARG, ZEB1, IRF8, BCL6, and TAL1 were increased and three TFs including RORC, RELA (p65), and IKZF2 were decreased in the glomeruli of LN. Specifically, DETFGs including RORC, RELA (p65) and IKZF2 were common differential transcription factors as they are downregulated in both the tubulointerstitium and glomeruli of LN (Table S2). We further searched for transcriptional cofactors and discovered four differential cofactors in the tubulointerstitium (CLU, NOTCH1, IKBKB, and RAF1) and five in the glomeruli (IKBKB, BTK, NOD2, S100A8, and MUC1; data not shown).

To confirm the importance of TFs and protein interactions, we analyzed differential transcription factor interactions with STRING and visualized PPI with Cytoscape. Modules cytohHubba has been used for sorting top hub TF genes by degree. Ranking DETFGs from highest to lowest by degree (from dark red to light yellow), the hub genes in the tubulointerstitium were STAT1, CEBPB, EGR1, and ETS1 (Figure 3D). STAT1 has the highest degree, suggesting its potential role in renal injury in the tubulointerstitium of LN. High-scored hub TF genes in the glomeruli were RELA (p65), RORC, BCL6, IRF8, and IKZF2 (Figure 3E). RELA (p65) has the highest degree in the glomeruli. We further analyzed differential transcription factors and cofactors in the glomeruli and tubulointerstitium together and demonstrated that the hub genes with the highest score were transcription factor STAT1 and cofactor NOTCH1 (Figure S1). These data suggest that transcription-related proteins, STAT1 and RELA (p65), may be associated with LN pathogenesis and development. The TFs of which the AUC was greater than 0.9 were STAT1, RORC, and EGR1 in the tubulointerstitium (Figure S2A), and PPARG and ZEB1 in the glomeruli (Figure S2B).

The correlation between CSE/CBS and DEGTFs (including STAT1 and RELA) in Glom and TubInt has been analyzed. The gene expression of CSE showed a negative relation to Hub TF STAT1 (Figure 3F–H).

### 3.4. RELA (p65) and STAT1 Were Increased in the Kidney Tissue of LN Patients

**Figure 4:**
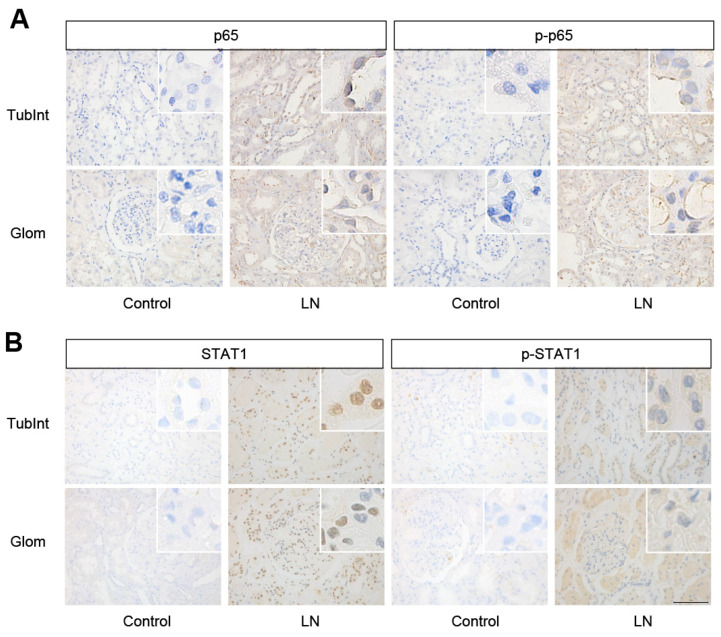
The expression of p65 and STAT1 in kidney tissue of LN patients. (**A**,**B**) Representative images of p65, p-p65, STAT1, and p-STAT1 staining in renal tissues of control and patients with LN (400×). TubInt, tubulointerstitium; Glom, glomeruli.

IHC was performed to detect the expression of Hub TFs in the renal tissues of LN patients. The results showed higher p65, p-p65, STAT1, and p-STAT1 protein expression in both the tubulointerstitium and glomeruli of patients with LN compared to that of normal controls (Figure 4A,B), suggesting that NF-κB and STAT1 are activated in the renal tissues of LN.

### 3.5. Key TFs in the Renal Tissue of Patients with LN Are Associated with T-Cell Activation and Function

**Figure 5:**
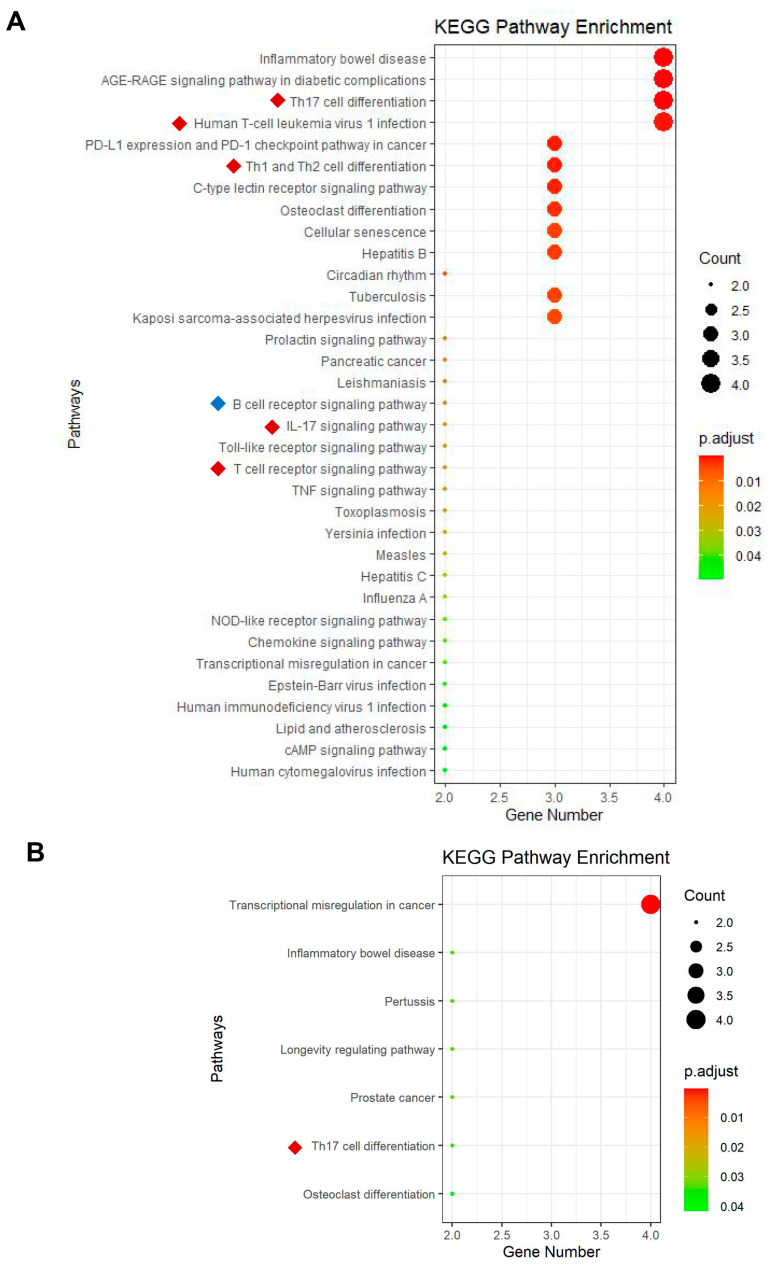
KEGG enrichment analysis of DETFGs in the tubulointerstitial and glomeruli of renal tissue of LN. (**A**) KEGG enrichment analysis of DETFGs in the tubulointerstitium; red and blue markers represent the signaling pathways associated with T cells and B cells, respectively. (**B**) KEGG enrichment analysis of DETFGs in glomeruli.

To explore the functions that differential transcription factors in LN mainly enriched, KEGG pathway enrichment analyses were performed by RStudio. In the tubulointerstitium, 34 pathways have been enriched based on 12 DETFGs. Five of these pathways are related to T-cell function or activation, and one pathway is connected to B cells (Figure 5A). In the glomeruli, seven pathways have been enriched based on eight DETFGs, of which one pathway is related to Th17 cell differentiation (Figure 5B). In general, KEGG enrichment of DETFGs is closely related to transcription and immunity, especially the function of T cells.

### 3.6. The Subtypes of Immune Cell Subtypes Are Differentially Regulated in the Renal Tissues of Patients with LN

**Figure 6:**
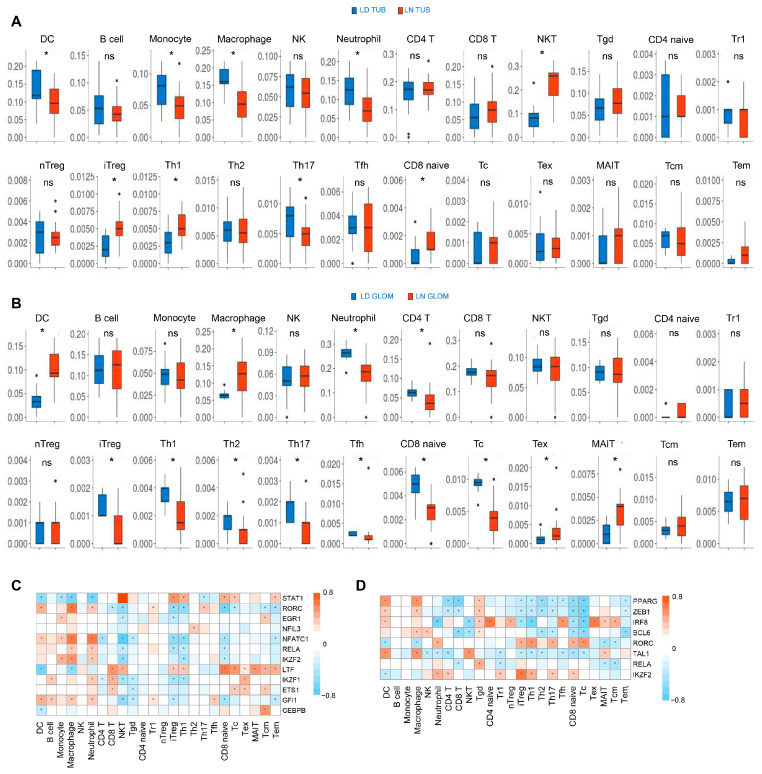
Immune cell subtype infiltration in the tubulointerstitial and glomeruli of renal tissue of LN. (**A**,**B**) The proportion of the 26 types of immune cell infiltration in the tubulointerstitium (**A**) and glomeruli (**B**). (**C**,**D**) Association between TFs and immune cell populations in LN. The association of TFs with immune cell populations in the tubulointerstitium (**C**) and glomeruli (**D**). Tgd, gamma delta T cells; Tr1, type 1 regulatory T cells; nTreg, natural T-regulatory cells; Tcm, central memory T; *, *p* < 0.05.

To evaluate the infiltration of immune cells and its relationship with transcription factors in LN, all genes expressed in the GSE32591 dataset have been analyzed in ImmucellAI. The GSE32591 dataset has more than 12,000 genes, which is much higher than that in the GSE113342 dataset, and a higher number of genes indicated a more comprehensive response to immune cell infiltration. In the tubulointerstitium, dendritic cells, monocytes, macrophages, neutrophils, and Th17 cells were downregulated, while NKT, induced T-regulatory cells (iTreg), Th1 cells, naive CD8+ T cells, and effector memory T cells (Tem) were upregulated in LN (Figure 6A). An increase of dendritic cells, macrophages, exhaustion T cells (Tex), and mucosal-associated invariant T cells (MAIT), and a decrease of neutrophils, CD4+ T cells, iTreg cells, Th1 cells, Th2 cells, Th17 cells, Tfh cells, CD8+ naive T cells, and CD8+ cytotoxic T cells (Tc) were observed in the glomeruli of LN (Figure 6B). Correlation analysis showed that transcription factors STAT1 and LTF, which increased in the renal tubulointerstitium, were positively correlated with T-cell subsets; in particular, STAT1 was significantly correlated with NKT (Figure 6C). The transcription factors that were positively associated with subsets of T cells in the glomeruli were IRF8, RORC, and IKZF2 (Figure 6D). As shown in Figure 6D, the differential TFs were also associated with a variety of other immune cell populations.

### 3.7. H2S Donor Alleviates Renal Injury, Hypersplenotrophy, and Arthritis in Pristane-Induced Lupus Mice

**Figure 7:**
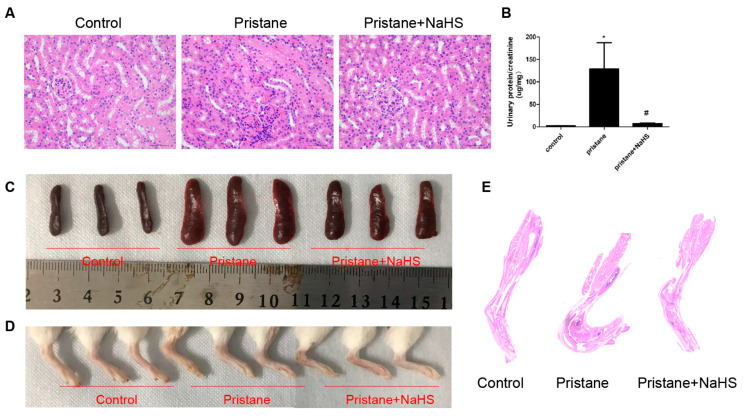
NaHS-attenuated pristane-induced renal injury, splenic proliferation, and arthritis. (**A**) Representative images of mouse renal tissues after HE staining (400×). (**B**) The ratio of urine protein and urine creatinine from mice was measured. Data are represented as the means ± SEM (*n* = 5). * *p* < 0.01, vs. control group. # *p* < 0.01, vs. pristane-induced lupus group. (**C**,**D**) Images of spleens and the lower ankle from control, pristane-induced with or without NaHS mice are shown. (**E**) Representative scan images of mouse lower ankle after HE staining.

Since hydrogen sulfide synthesis is insufficient in the kidney of LN patients and mice, a well-known H2S donor, NaHS, was used to investigate whether H2S prevents lupus activity in pristane-induced lupus mice. The remarkable renal pathological injury was observed by HE staining in pristane-infused mice (Figure 7A). The ratio of urine protein and creatinine was reduced after NaHS injection in pristane-infused mice (Figure 7B). Meanwhile, the treatment of NaHS improved joint swelling and spleen proliferation in pristane-induced lupus mice (Figure 7C–E).

### 3.8. H2S Donor GYY4137 Attenuates Renal Injury and Reverses Reduced CSE and CBS Expression in MRL/lpr Mice

**Figure 8:**
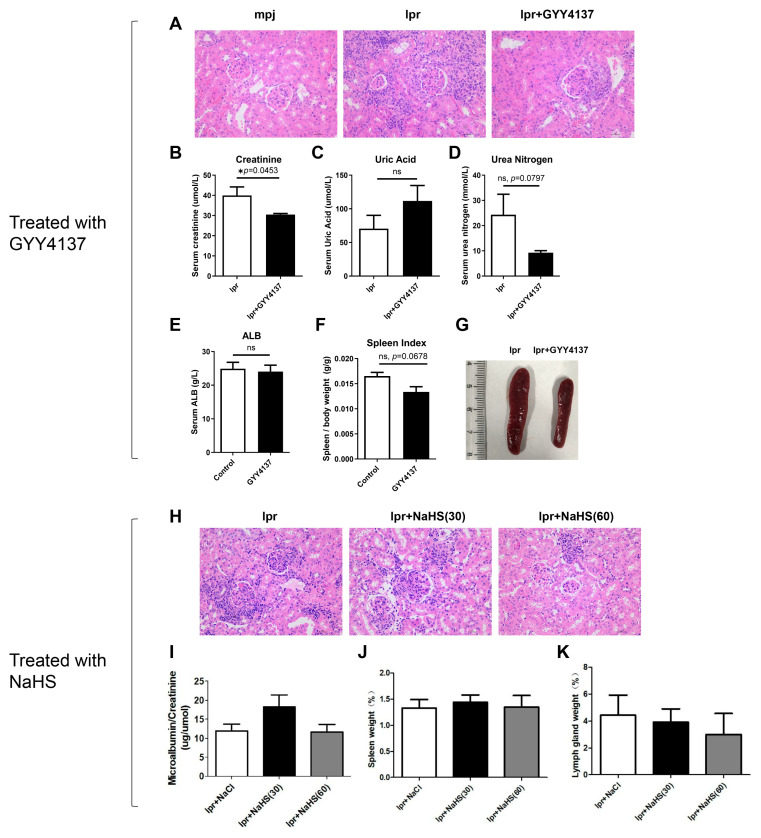
The H2S donors, GYY4137 and NaHS, partially attenuated renal injury in MRL/lpr mice. (**A**) Representative images of mouse renal tissues after HE staining in GYY4137-treated mice (400×). (**B**–**E**) The serum levels of creatinine, urea nitrogen, uric acid, and albumin were detected upon GYY4137 injection. (**F**,**G**) The ratio of spleen weight and body weight and representative images of the spleen have been shown. Data are represented as the means ± SEM (*n* = 6). * *p* < 0.05. (**H**) Representative images of mouse renal tissues after HE staining in NaHS-treated mice. (**I**) The ratio of urine protein and urine creatinine from mice was measured. (**J**,**K**) The ratio of spleen or cervical lymph node weight and body weight was calculated.

To verify the therapeutic effect of exogenous H2S in LN, we used the H2S donors, GYY4137 and NaHS, to treat MRL/lpr lupus mice. In GYY4137-treated mice, inflammation around the glomerulus was significantly improved in renal HE staining (Figure 8A). In biochemical blood tests, we found GYY4137 also decreases serum creatinine levels in MRL/lpr mice (Figure 8B); however, indicators such as serum urea nitrogen, albumin, and splenomegaly showed no significant difference (Figure 8C–G). In NaHS-treated mice, renal inflammation seemed to result in a reduction in HE staining and a tendency to decrease in the weight of lymph nodes, but the urine protein creatinine ratio and spleen weight were not ameliorated (Figure 8H–K).

### 3.9. H2S Donor Recovers Low Expression of CSE and CBS

**Figure 9:**
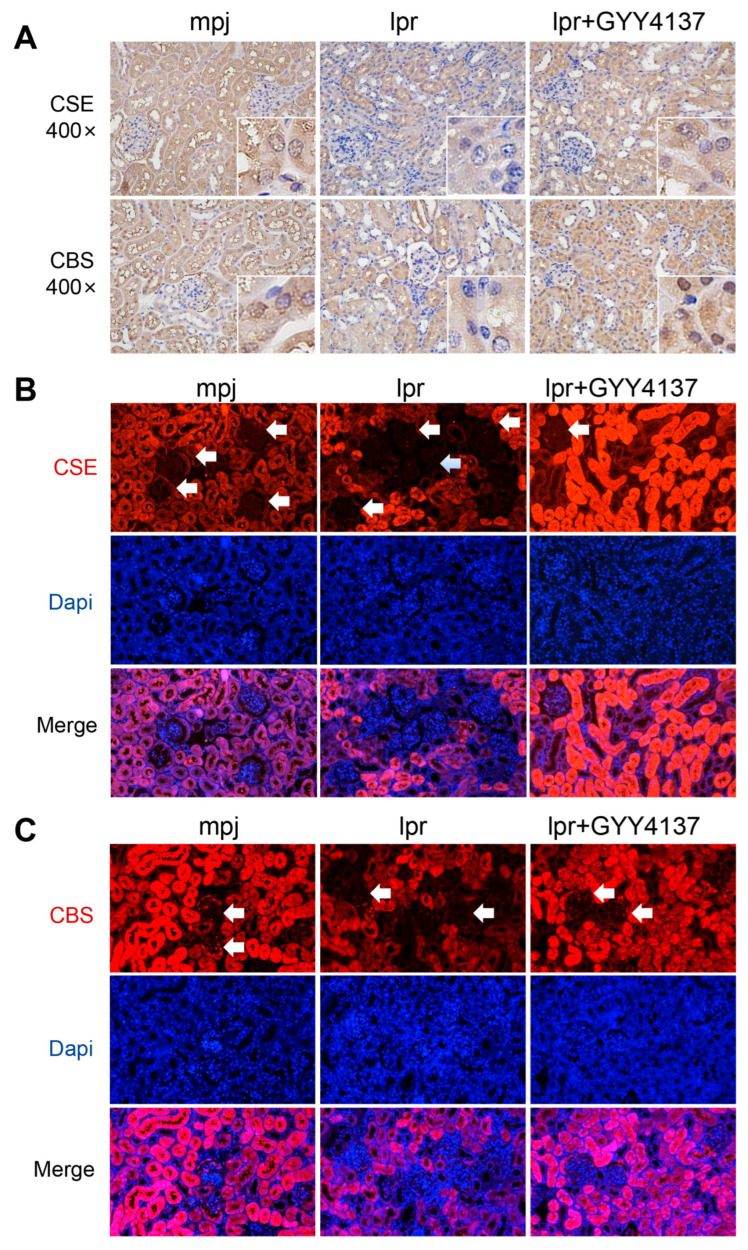
H2S donor recovers the reduced expression of CSE and CBS in MRL/lpr mice. (**A**) Representative images of mouse renal tissues after IHC staining of CSE and CBS in MRL/lpr and MRL/mpj mice (400×). (**B**,**C**) Representative images of mouse renal tissues after IF staining of CSE, CBS, and DAPI in MRL/lpr and MRL/mpj mice (200×); the white arrow refers to the glomerulus.

To explore the mechanism of the exogenous H2S-supplement-conferred attenuation of LN, we measured the expression of CSE and CBS by IHC staining in MRL/lpr mice. The H2S donor GYY4137 increased the expression of CSE and CBS in both the tubulointerstitium and glomeruli (Figure 9).

### 3.10. H2S Donors Inhibit p65 and p-p65 Expression and Reduce Inflammatory Cell Infiltration in the Kidney of Lupus Mice

**Figure 10:**
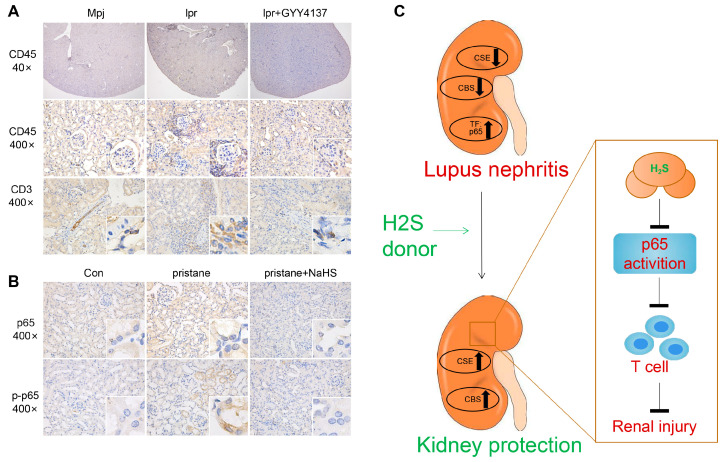
H2S supplementation reduces the expression of p65 and T-cell infiltration in the kidney of lupus mice. (**A**,**B**) Representative images of mouse renal tissues after IHC staining of p65 and p-p65 in pristane-induced mice (400×). (**C**) A model depicting the potential role of the H2S donor in the attenuation of LN.

Furthermore, GYY4137 significantly reduced CD45-positive inflammatory cells (periglomerular) and CD3-positive T cells (perivascular) infiltrated in the kidney tissue of lpr mice (Figure 10A). Moreover, we found that the downregulated expression of p65 and p-p65 were increased by pristane infusion, but were suppressed by subsequent NaHS treatment (Figure 10B). A graphical illustration for the possible mechanisms underlying the role of H2S in LN has been shown in Figure 10C.

## 4. Discussion

There are four central and novel findings of this study: 1. Hydrogen sulfide synthase, CBS and CSE, was downregulated in renal tissues from patients and mice with LN, and the expression of CBS and CSE is related to the renal damage index in LN patients. 2. H2S donors partially rescue renal injury in lupus mice. 3. STAT1 and RELA (p65) are hub differential TFs between LN and normal kidneys, and the differential TFs in lupus renal tissues were mainly enriched in T-cell-related signaling. 4. H2S supplementation significantly attenuated the expression of transcription factor p65 and reduced the infiltration of renal inflammatory cells in the kidney of lupus mice. Collectively, these composite results revealed that H2S donors alleviate lupus kidney injury by reducing the expression of the renal T-cell-related pro-inflammatory TF p65, which is a potential new therapeutic drug option in LN.

Of note, the present study showed that the expression pattern of CBS and CSE were not consistent in three animal models of lupus. There was no difference in the CBS and CSE mRNA expression between pristane and control mice. CBS mRNA expression was not significantly changed in the R848 model. In contrast, CBS and CSE mRNA expression was significantly reduced in MRL/lpr mice. In fact, less severity of renal damage was found in the pristane model compared with MRL/lpr mice. Since we found that CSE and CBS expression levels were negatively associated with the disease severity of the patients with LN, it is not excluded that the CSE and CBS expression pattern in the pristane model might be associated with a lower severity of renal damage.

The decline in H2S level has been reported in numerous renal disorders, such as diabetic nephropathy [30]. In animal models of renal diseases, treatment with H2S donors could restore H2S levels and improve renal functions by preventing oxidative stress, inflammation, and necrosis by reducing superoxide formation, lipid peroxidation, iNOS, NF-κB, TNF-α, and malondialdehyde levels [24,30]. Han Y et al. reported hydrogen sulfide inhibits the abnormal proliferation of lymphocytes via the AKT/GSK3β signal pathway in systemic lupus erythematosus patients [31]. Supplementing with hydrogen sulfide also has a therapeutic effect on autoimmune diseases including rheumatoid arthritis [32]. The present study demonstrated the H2S donors alleviated the kidney injury in three lupus mouse models. It has been demonstrated that H2S can suppress NF-κB activation [33]. Interestingly, we showed that p65 is one of the hub TFs in LN pathogenesis. Treatment of lupus mice with H2S donors reduced the active NF-κB form p-p65 level as well as immune cell infiltration in renal tissues, which further confirms the therapeutic effects of H2S donors for LN. Of note, the expression levels of H2S-producing enzymes can be enhanced by an H2S supplement [34,35]. Consistently, we found that an H2S donor could increase CSE and CBS expression in the renal tissues of lupus mice. These data indicate that the therapeutic effect of H2S donors on renal damage is partly through promoting endogenous H2S production.

Renal transcription factors are involved in lupus nephritis injury by regulating the expression of a large number of downstream target genes. Through enrichment analysis, we found that these differential TFs were mainly related to the innate immune response, inflammatory response, maturation and differentiation of T cells, B-cell receptor differentiation, leukocyte migration, and cell adhesion. These factors are closely related to the pathogenesis of LN [36,37]. T-cell-related pathways were the most common among the enriched pathways in our study. Numerous T-cell subsets play distinct roles in promoting systemic autoimmunity and end-organ damage in lupus. Tfh cells enable autoantibody production, and Th17 subsets promote inflammation, while defects in Treg cells lead to loss maintain self-tolerance by suppressing autoreactive lymphocytes [38,39]. A selective dysregulation of Th17 cells and Treg/Th17 balance substantially advanced the understanding of lupus [40,41]. Combined with our results, it suggested that differentially expressed transcription factors may be involved in the pathogenesis of lupus by affecting T-cell function. Furthermore, the interaction between transcription factors is essential in regulating gene expression, and there is evidence that the risk allele of rs6590330 is associated with decreased ETS1 expression and increased SLE risk by enhancing the binding of p-STAT1 [42]. Our present study further demonstrated that STAT1 and ETS1 were hub TFs in the tubulointerstitium, which provided evidence for the interaction of TFs in lupus.

Previous studies have shown that T cells and p65 are involved in the pathogenesis of lupus, while hydrogen sulfide suppresses inflammation in diseased kidneys. Our data further confirmed that H2S donors retrieved renal T-cell infiltration and p65 expression in mice with lupus nephritis, which suggested H2S may serve as a therapeutic target for LN. In addition, other T-cell inflammatory-associated transcription factors were also identified in the present study and deserve further investigation. Renal T cells have a crucial function in immune tolerance regulation and are one of the most critical research hotspots for autoimmunity diseases, especially lupus nephritis [43,44]. Here, we also observed the differential expression of immune T cells including NKT, iTreg cells, Th1 cells, Th2 cells, Th17 cells, Tfh cells, Tem, CD4+ naive T cells, CD8+ naive T cells, Tc, Tex, and MAIT in LN. Various immune cells can infiltrate kidney tissues; single-cell data showed that the infiltrating in the human kidney of LN are T cells and macrophages [45,46]. Activated T cells, on the one hand, affect B-cell activation and antibody production, and, on the other hand, infiltrate kidney tissues and secrete cytokines, causing kidney damage. We identified two transcription factors (STAT1 and LTF) that were positively correlated with T-cell subset infiltration in the lupus tubulointerstitium, and three transcription factors (IRF8, RORC and IKZF2) that were positively associated with T-cell subset infiltration in the glomeruli. The association of STAT1 and IRF8 with T cells in immune and inflammatory responses has been extensively studied [47,48]. However, the role and mechanisms of these TFs in T-cell infiltration and function during LN remain largely unknown and could be an interesting topic for future study.

## 5. Conclusions

The present study demonstrated that CSE and CBS expression was significantly downregulated in the renal tissues of SLE patients with LN and animal models of SLE. RELA and STAT1 were the hub genes of TFs in LN patients. Both of RELA and STAT1 factors were activated in the renal tissues of LN patients. H2S donors alleviated renal injury and inhibited RELA activation and immune cell infiltration in the renal tissues of animal models of SLE. Our data indicate that the CSE/CBS/H2S system contributes to LN progress. Supplementation of H2S would be a potential therapeutic strategy for LN.

## Data Availability

The datasets presented in this study can be found in online repositories. The names of the repository/repositories and accession number(s) can be found in the article or Supplementary Data.

## Supplementary Materials

The following supporting information can be downloaded at: https://www.mdpi.com/article/10.3390/biology12020318/s1, Supplemental Figure S1. The interaction of hub transcription factors and transcription cofactors in the tubulointerstitium and glomeruli. Supplemental Figure S2. ROC curve of DETFGs in GSE32591. Supplemental Table S1. Human TFs and human coTFs. Supplemental Table S2. Differential TFs in renal tissue of patients with LN and healthy living donors.
