# Supplementary material for: Reduced Renal CSE/CBS/H2S Contributes to the Progress of Lupus Nephritis
Source: Biology (Basel). 2023 Feb 16;12(2):318. doi: 10.3390/biology12020318 (PMC9953544; doi:10.3390/biology12020318)

**[Supplement](javascript:;)al Figure 1. The interaction of hub** **transcription factors and transcription cofactors in the tubulointerstitium and glomeruli.** A. The redder the color, the higher the degree through modules cytohHubba for sorting top hub genes.


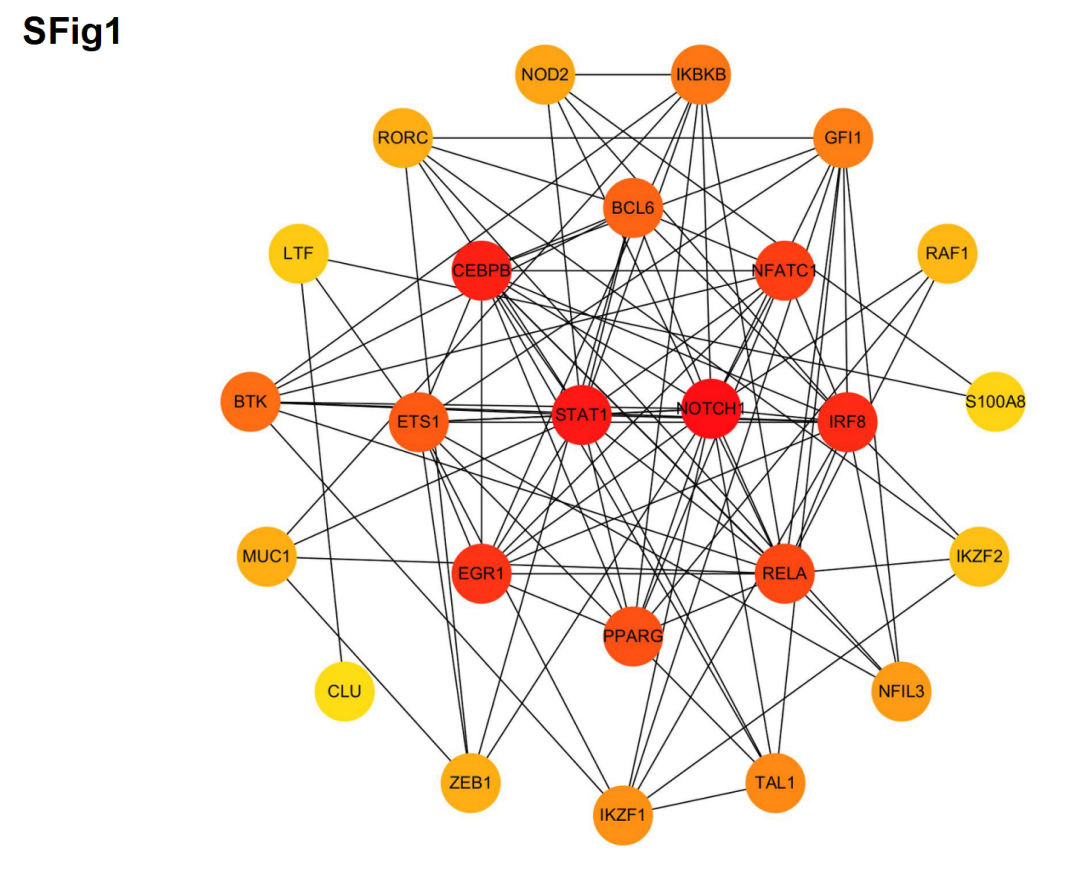


**[Supplement](javascript:;)al Figure 2.** **ROC curve of DETFGs in GSE32591.** (**A**) The ROC curve and AUC of transcription factors in the tubulointerstitium. (**B**) The ROC curve and AUC of transcription factors in the glomeruli.


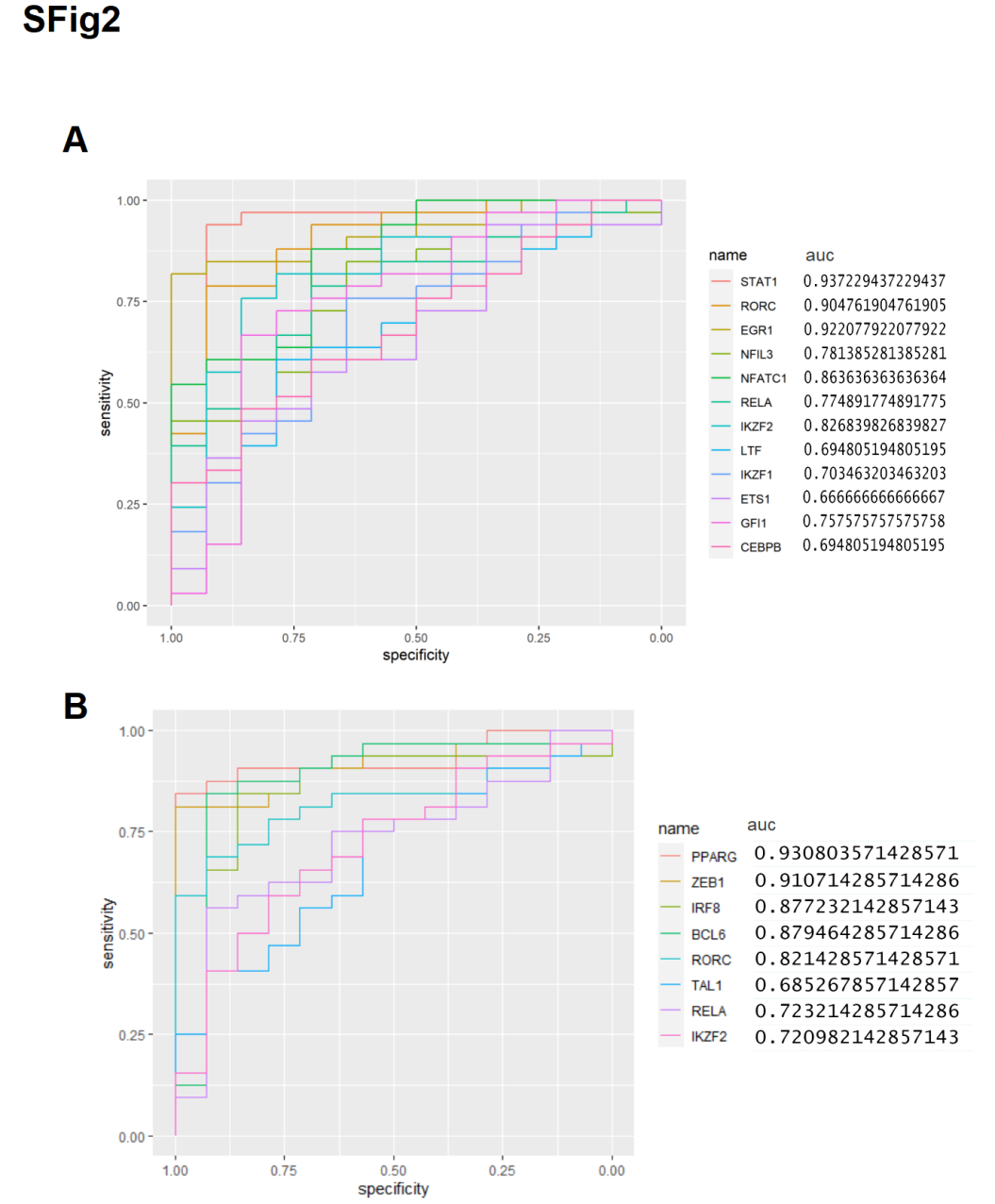

Supplement: Supplementary file 1 [file biology-12-00318-s001.zip › Sfig1-2.docx]
